# Comparative study of stunting measurement in children using WHO procedure and Growth Length Mat in Ghana

**DOI:** 10.1186/s13104-022-06259-x

**Published:** 2022-12-21

**Authors:** Nafisatu Bukari, Alhassan Danaa, Abdulai Mubarak, Wilfred W. Forfoe, Ayishetu Gariba, Zakari Ali

**Affiliations:** 1 School of Human Nutrition, McGill University, Ste Anne-de-Bellevue-Montreal, Geneva, QC Canada; 2 Nutrition Unit, Tain District Hospital, P. O. Box 100, Tain, Bono Region Ghana; 3 Nutrition Unit, Nanton Health Directorate, P. O. Box 45, Nanton, Northern Region Ghana; 4 Department of Nutrition for Health, Development, World Health Organization, Geneva, Switzerland; 5 Student Affairs department, C. K. Tedam University of Technology and Applied Sciences (C. K. T. - UTAS), P. O. Box 24, Navrongo, Ghana; 6 Nutrition and Planetary Health Theme, London School of Hygiene and Tropical Medicine, MRC Unit The Gambia, Atlantic Boulevard, Fajara, P. O. Box 273, Banjul, The Gambia

**Keywords:** Growth, Height, Infant, Stunting, Length mat

## Abstract

**Objective:**

Height data is not useful immediately without further processing. The Growth Length Mat (Stunting Mat) was designed to stunting directly. However, the Mat has not been validated for use in Ghana. This study compared stunting measured with the Mat and stunting measured following the WHO recommended procedure. We sampled 163 children aged 6–24 months in the Bono region of Ghana. We also explored the acceptability and interpretability of the two procedures among mothers and healthcare givers.

**Results:**

The prevalence of stunting was 3.7% and 11.7% using the Mat and WHO procedures respectively. The Growth Length Mat had very low sensitivity (21.0%) but high specificity (98.6%) of detecting stunting in children. However, in younger aged children, the Mat was more accurate in detecting stunting. Both healthcare workers and caregivers found it easy to interpret the stunting status of children using the Mat. We conclude that the Growth Length Mat was less sensitive at detecting child stunting compared to the current gold standard of measuring stunting in younger children. There are possibilities to improve the accuracy and utility of the Mat for measuring stunting in low-resource settings by re-designing the mat to be more age appropriate.

**Supplementary Information:**

The online version contains supplementary material available at 10.1186/s13104-022-06259-x.

## Introduction

A child is classified as stunted when they are too short for their age [1]. Based on international agreement, children are considered stunted if their length/height-for-age is less than –  2 SDs from the World Health Organization (WHO) Child Growth Standards median for the same age and sex [2, 3]. Poor health conditions coupled with nutritional inadequacy often result in stunted growth which negatively affects overall health outcomes [4-6]. Stunted children are more likely to suffer irreversible cognitive and physical health damages that could be lifelong and even be passed on to the next generation [7]. Stunting also has negative long-term educational and economic consequences [1, 7]. Stunting usually goes unnoticed in most community settings particularly among populations where short stature is common. Stunting may also go unnoticed because height is not routinely measured as part of growth monitoring [4]. The prevalence of stunting in under five children in Ghana decreased from 35.5 to 17.5% from 2003 to 2017 [8] respectively. Similarly, the stunting in the Bono Ahafo region of Ghana decreased from 25.2 to 17.2% within the same period [6, 9].

Height/length of children is measuring as part of a WHO procedure for determining growth deficits in children in routine national surveys. However, these measurements require further processing in reference to age and sex to determine the degree of stunting [10]. The Growth Length Mat also called descriptively as “Stunting Mat or Child Length Mat” is an innovative tool initially developed in Bolivia and adapted by a USAID funded project (NOURISH) in Cambodia to support community and health workers to directly detect stunting and rapidly identify children who may need attention [11]. The mat seeks to address the need for special skills and processing needed to use the WHO procedure in determining child stunting [11].

The WHO procedure normally involves using a calibrated wooden/plastic board with a headpiece and a movable foot piece which is used for measuring height or length of children (0–59 months of age). Its foot piece can also serve as a headpiece when measuring the standing height of children and compared with WHO standard growth charts [12]. The mat on the other hand is 96 cm long and 50 cm wide [11]. It is made with a flexible and foldable material and relatively light to carry. The mat is colour coded to indicate the status of a child (normal or stunted) when placed on it depending on the sex and age and so measures child stunting directly.

The objective of this study was to compare the use of the WHO procedure and Growth Length Mat in assessing stunting in children 6–24 months in one district of the Bono region of Ghana.

## Main text

### Materials and methods

#### Study design, population, and sampling

The study design was cross-sectional and both WHO procedure and Growth Length Mat were used to measure the stunting status of children. It was conducted in the Tain District of Bono region of Ghana. The study participants were sampled from Tain District Hospital and other CHIP zones under the hospital. Simple random and convenient sampling methods were adopted to select facilities and respondents for the study respectively. Primary targets were with children aged 6–24 months. The study sampled 163 participants, consisting of mothers/caregivers with children between the ages of 6 to 24 months of age who were attending child welfare clinic (CWC). Child health record books were used to determine the children who qualified to be part of the study at each of the health facility visited. Mothers were shown how both an infantometer and Growth Length Mat are used, and health workers were given detailed training as to how to use the tools and the conversion heights/lengths in reference to age to generate z-scores. We also administered a short semi-structured questionnaire which elicited data on socio-demographic information, the ease of using the WHO procedure and the mat. Finally, we asked health care providers about their experiences and challenges with using both procedures in determining child stunting.

#### Measurement of stunting using the growth length mat

Stunting status with the mat was measured using the mat designed by SPRING USAID Ghana in collaboration with GHS. The mat has two colour codes (red indicating stunted and green indicating normal) with age ranges 6, 12, and 18 months for both sexes (See illustration in Additional file 1). Children were measured by laying the mat (according to their age and sex) in a recumbent position with minimal clothing acceptable by local culture. Measurements were done by two assessors together assisted by mothers/caregivers when requires with the child’s eye looking straight up similar to measuring standard length [3]. Each child was measured twice with the mat and the most appropriate status was recorded.

#### Measurement of stunting following WHO procedure

Recumbent length was measured with a wooden board. Wooden board was placed on flat surface and measurements were taken. Each child was measured twice by two measurers, one who was responsible for positioning the child and another who recorded the measurement. The average was taken to represent the length. While the Growth Length Mat was used to determine the stunting status of children on the field, the WHO procedure was used to measure the length (cm) of children and later converted to height-for-age Z-scores (HAZ) using WHO-anthro software.

#### Data analysis

Data collected from the field was entered and analyzed using Stata 16 (Stata Corp, USA). Study results were presented in tables. The WHO-anthro was used to generate length-for-age Z-scores (HAZ) of children. The Z-scores were transformed into stunting status. Stunting was defined as HAZ <-2.

## Results

A slightly higher number of children included in our analysis were male (58.3%) with a good age distribution (Table 1).

**Table 1:** Background characteristics and stunting status of children sampled

Characteristic	Frequency	Percent
Sex		
Male	95	58.3
Female	68	41.7
Age group (months)		
6–9	48	29.4
10–12	37	22.7
13–15	25	15.3
16–18	26	16.0
19–24	27	16.6

Overall, using the WHO existing gold standard for classifying child stunting, 12% of the children were stunted compared to 4% detected using the Growth Length Mat (Additional file 2). While detecting only a small number of stunted children, the mat tended to be quite effective at detecting stunting in younger children (6–9 months) than older children beyond 9 months. Again, despite poor detection capability of the mat, it was quite useful at detecting stunting in female children than in male children and younger children (between 6 and 9 months) (Table 2). Measures of test validity showed that, compared to the WHO gold standard of measuring stunting in children, the mat had only 21.0% sensitivity and 98.6% specificity (Table 3). Meaning that, the mat could only detect 21.0% of real stunted children but when children were not stunted, the mat was 98.6% more likely to detect that they were not stunted. Consequently, the mat had 66.7% and 90.4% positive predictive value and negative predictive value respectively (Table 3). The measurement of the stunting status of children using the mat came with several challenges. Among which were difficulty correctly positioning children on the mat and reading values on the mat but both health workers and caregivers widely accepted the mat and found the colour codes depicting stunting status easy to interpret. Comparatively, health workers expressed more difficulty measuring stunting status following the WHO procedure. Most health workers (50%) agreed it was cumbersome to measure the length or height of children using the height board which requires special skills and at least two assessors. The combination of age and height to generate z-scores to determine stunting status using special software such as WHO-anthro was also a challenge (Additional file 3).

**Table 2:** Distribution of stunting prevalence by age group between WHO procedure and the Growth Length mat

Age group (months)	WHO procedure	Length mat
	Stunted	Stunted
	n(%)	n(%)
6–9	5(10.4)	4(8.3)
10–12	3(8.1)	0(0.0)
13–15	3(12.0)	0(0.0)
16–18	4(15.4	2(7.7)
19–24	4(14.8)	0(0.0)
Total	19(11.7)	6(3.7)
Sex of child		
Male	14(14.7)	3(3.2)
Female	5(7.3)	3(4.4)
Total	19(11.7)	6(3.7)

**Table 3:** Test validity measurements

		WHO procedure/gold standard		Total
		Stunted	Not stunted	
Length Mat/ new test	Stunted	a (4)	b (2)	6
	Not stunted	c (15)	d (142)	157
	Total	19	144	163

Sensitivity = a/(a + c) = 4/(19) = 21.0%

Specificity = d/(b + d) = 142/144 = 98.6%

Positive predictive value (PPV) = a/(a + b) = 4/6 = 66.7%

Negative predictive value (NPV) = d/(c + d) = 142/157 = 90.4%

## Discussion

The Growth Length Mat was designed to measure the stunting status of children under 2 years of age directly, without the more complicated WHO procedures that involve considerable training, a combination of processes, and multiple measurements. The mat was initially designed in Bolivia and a validated prototype adapted for use in Cambodia by the NOURISH project to help screen children for stunting. The initial validation work in Cambodia produced similar results to our current study [13]. However, our study found far less sensitivity of the mat compared to Cambodia (21.0% vs. 57.0% for Ghana and Cambodia respectively) [13]. In Ghana, this tool was adopted by the Strengthening Partnership, Results, and Innovations in Nutrition Globally (SPRING) project for stunting screening without validation. Hence this research was conducted to evaluate the level of accuracy of the mat in detecting stunting compared to the standard WHO procedure. According to the recent demographic and health survey of Ghana, the prevalence of stunting decreased from 23–19% nationwide and the prevalence in the Bono region declined from 25.2 to 17.2 between 2008 and 2014. Our results show that the prevalence of stunting in children 6–23 months in the Tain district of Bono region is 11.7% which is consistent with the regional average. Like weight measurements, measuring heights/lengths is an important part of growth monitoring of children but is less utilized by health workers in Ghana due to difficulties associated with measuring height which also requires two people to measure. Altobelli et al. 2020 [14] demonstrated that community health workers with trainings on height/length measurement felt motivated and positive and demonstrated a high level of knowledge in performing tasks compared to the control group. The infantometer is made with wooden material with both movable head and foot pieces with graduations indicated. The tool measures the length/height of children and compared them with the child’s age to calculate z-scores to determine the stunting status of children. The Growth Length Mat is made with a flexy material with colour-coding indicated on mat for the sex of children and their age ranges. This design is similar to the Mid Upper Arm Circumference (MUAC) tape developed by UNICEF [15]. Hence, it is easier to read by caregivers as there is no need to compare with any variable before determining the stunting status of the child.

The mat has 6 months age range that is 6, 12, and 18 months sections. For example, 6–11 months old children are grouped and children 12–17 months old also measured together as a group according to sex. Therefore, stunted, but relatively older children within a group are more likely to touch the “normal line”, while normal but younger children of in a group are less likely to touch the “normal line”. Overall, the mat was very poor in detecting stunting in children over 12 months old. While the idea for a simple and easy tool to measure stunting is warranted, the mat will need substantial improvements in its design to improve its accuracy in detecting stunting in children before widespread adoption across populations. One way to improve upon the design of the version of the mat used in Ghana could be a simple inclusion of the profile of the infant/child to make sure the child is correctly positioned.

We can conclude that the current design of the Growth Length Mat used in Ghana is less sensitive in detecting child stunting compared to the WHO gold standard of measuring stunting in children. There are possibilities to improve the accuracy and utility of the Growth Length Mat for measuring stunting in low-resource settings by re-designing with positioning illustrations or building narrower age-appropriate mats.

### Limitations

- Due to lack of repeated values of measurements made, we could not compute reliability measures of the tests.
- Despite training of our field staff and engaging two assessors per measure, it is still likely that some measurements of length of children were less accurate due to difficulty in placing younger children on the infantometer or Growth Length Mat which can affect the validity of our results.

## Supplementary information

**Additional file 1: Figure S1.** The Growth Length Mat 

**Additional file 2: Figure S1. **Comparability of stunting prevalence by WHOprocedure and Growth Length Mat

**Additional file 3: Table S1. **Interpretability andacceptability of Growth Length Mat 

## Data Availability

All data supporting the conclusions of this study are included in the manuscript.

## Abbreviations

CWC: Child Welfare Clinic
GDHS: Ghana Demographic and Health Survey
MUAC: Mid-Upper-Arm Circumference
SPRING: Strengthening Partnership, Results, and Innovations in Nutrition Globally
