# Supplementary material for: Comparative study of stunting measurement in children using WHO procedure and Growth Length Mat in Ghana
Source: BMC Res Notes. 2022 Dec 21;15:374. doi: 10.1186/s13104-022-06259-x (PMC9768933; doi:10.1186/s13104-022-06259-x)
Supplement: Supplementary file 1 — Additional file 1: Figure S1. The Growth Length Mat [file 13104_2022_6259_MOESM1_ESM.docx]

**Comparative study of stunting measurement in children using WHO procedure and Growth Length Mat in Ghana**

**Supplementary material**


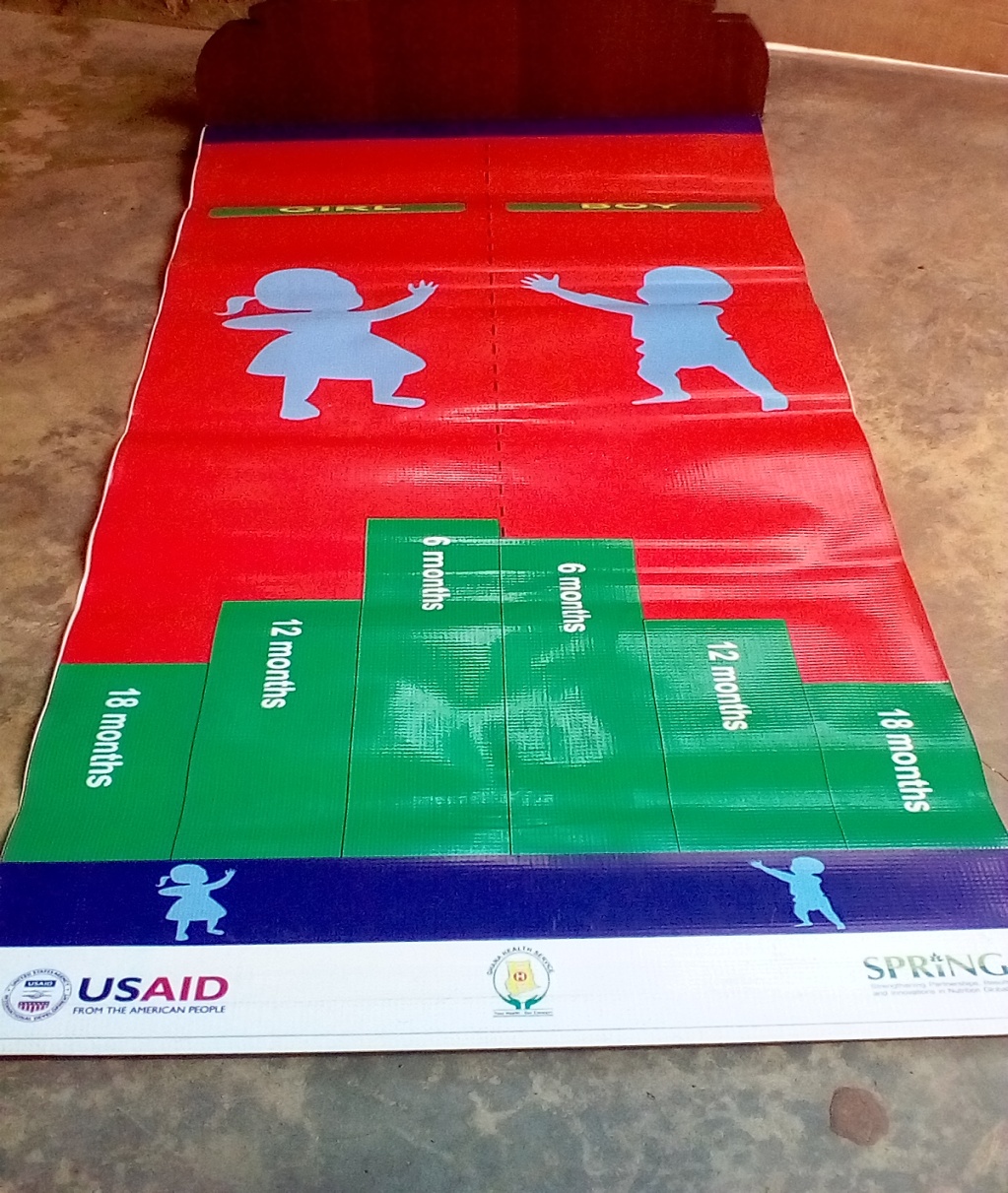


**Figure S1: The Growth Length Mat**
