# Supplementary material for: Comparative study of stunting measurement in children using WHO procedure and Growth Length Mat in Ghana
Source: BMC Res Notes. 2022 Dec 21;15:374. doi: 10.1186/s13104-022-06259-x (PMC9768933; doi:10.1186/s13104-022-06259-x)
Supplement: Supplementary file 3 — Additional file 3: Table S1. Interpretability andacceptability of Growth Length Mat [file 13104_2022_6259_MOESM3_ESM.docx]

**Comparative study of stunting measurement in children using WHO procedure and** **Growth Length Mat in Ghana**

**Supplementary material**

**Table S1: Interpretability and acceptability of Growth Length Mat**

| Difficulties | WHO procedure | | Length mat | |
| --- | --- | --- | --- | --- |
|  | Frequency | % | Frequency | % |
| Difficulty laying a child on the tool | 7 | 43.7 | 3 | 18.8 |
| Difficulty reading from tape | 1 | 6.3 | 0 | 0 |
| Difficulty comparing with age | 2 | 12.5 | 0 | 0 |
| Graduations not indicated | 0 | 0 | 1 | 6.3 |
| No difficulty at all | 6 | 37.5 | 12 | 73.0 |
